# Cell wall integrity is compromised under temperature stress in *Schizosaccharomyces pombe* expressing a valproic acid-sensitive vas4 mutant

**DOI:** 10.1038/s41598-021-92466-8

**Published:** 2021-06-29

**Authors:** Sen Qiao, Xiaofang Luo, Hui Wang, Yue Fang, Lili Zhang

**Affiliations:** 1 Department of Laboratory Medicine, The Affiliated Hospital of Zunyi Medical University, Zunyi, 563003 People’s Republic of China; 2 School of Laboratory Medicine, Zunyi Medical University, Zunyi, 563003 People’s Republic of China; 3 Department of Microbial and Biochemical Pharmacy, School of Pharmacy, China Medical University, Shenyang, 110112 People’s Republic of China

**Keywords:** Biochemistry, Enzymes, Proteins

## Abstract

Valproic acid (VPA) is widely used as a eutherapeutic and safe anticonvulsant drug, but the mechanism is not well elucidated. Histone deacetylases (HDACs) were first identified as direct targets of VPA. Many loss-of function mutants in *S. pombe* have been shown to be VPA sensitive but not sensitive to other HDAC inhibitors, such as sodium butyrate or trichostatin A (TSA). This difference suggests that there are multiple VPA target genes. In the current study, we isolated a VPA-sensitive (*vas*) mutant, *vas4-1*, and cloned the VPA target gene *vas4*^+^/*vrg4*^+^ by performing complementation experiments. The *vas4*^+^/*vrg4*^+^ gene encodes a putative Golgi GDP-mannose transporter, Vrg4, which is highly homologous with ScVrg4p. Physiological experiments indicated that SpVrg4p is involved in maintaining cell wall integrity (CWI) under high- or low-temperature stress. The results of a coimmunoprecipitation assay suggested that SpVrg4p may be transferred from the ER to the Golgi through SpGot1p loaded COPII vesicles, and both single and double mutations (S263C and A271V) in SpVrg4p compromised this transfer. Our results suggested that CWI in *S. pombe* is compromised under temperature stress by the VPA-sensitive *vas4* mutant*.*

## Introduction

Valproic acid (VPA) is a short-chain fatty acid that is widely used as an anticonvulsant for the treatment of various types of epilepsy and seizures, such as absence, myoclonic, generalized and partial seizures^1–3^ and is presumed to target GABA transaminase, succinate semialdehyde dehydrogenase, alpha-ketoglutarate dehydrogenase and Na^+^ channels^4,5^. It has also been proposed to be a therapeutic for certain types of cancer, Alzheimer’s disease, and HIV treatment^6–9^. However, the mechanisms of its therapeutic effect remain to be elucidated.

To gain further insights into the molecular mechanisms of VPA drug action, we developed a genetic screen for mutants that show hypersensitivity to VPA in the fission yeast *Schizosaccharomyces pombe*. The first identified gene in this screening was *vas1*^+^/*vps45*^+^, encoding a homologue of human *VPS45* and *S. cerevisiae VPS45*^10^. *Vas2-1* is an allele of the *aps1*^+^ gene that encodes the σ subunit of the AP-1 complex^11^, and *vas3-1* is an allele of the *ric1*^+^ gene that encodes a homologue of budding yeast *RIC1*^12^. A genome-wide screen of 3004 haploid deletion strains confirmed 148 VPA-sensitive deletion strains; these mutants are involved in regulating DNA and RNA metabolism, signal transduction, membrane trafficking, chromatin remodelling, ubiquitination*, *etc.^13^.

In the current study, we isolated the VPA-sensitive (*vas*) mutant *vas4-1* and cloned the VPA target gene *vas4*^+^/*vrg4*^+^ through complementation experiments. *vas4*^+^/*vrg4*^+^ encodes a putative transmembrane Golgi GDP-mannose transporter protein that is highly homologous with ScVrg4p. In budding yeast, Vrg4p is essential for cell wall integrity (CWI) and normal Golgi function, such as protein glycosylation and sphingolipid mannosylation, and the null mutant is lethal^14,15^. Here, we found that the *vas4*^+^/*vrg4*^+^ gene was important for maintaining CWI under temperature stress in fission yeast. A biochemical assay suggested that trafficking of Vrg4p from the ER to the Golgi might be realized through Got1p-loaded COPII vesicles. Our results suggested that the *vas4*^+^/*vrg4*^+^ gene is a novel candidate VPA target, and mutations at S263 and A271, which are located in the nucleotide sugar-binding motif, compromised Vrg4p function and abolished its interaction with Got1p.

## Methods and materials

### Media and genetic and molecular biology methods

The complete YPD medium and standard genetic and recombinant-DNA methods have been described previously^10,16^.

### Isolation of the ***vas4-1*** mutant and cloning of the ***vrg4***^+^ gene

The *vas4-1* mutant was identified through a screening of cells that had been mutagenized with nitrosoguanidine as described previously^17^. To clone the *vrg4*^+^ gene, the *vas4-1* mutant was grown at 27 °C and transformed with an *S. pombe* genomic DNA library constructed in the vector PKB109^18^. Leu^+^ transformants were replica plated onto YPD plates containing VPA at 27 °C, and the plasmid DNA was recovered from transformants that showed plasmid-dependent rescue. These plasmids complemented the VPA sensitivity of the *vas4-1* mutant. By DNA sequencing, the suppressing plasmids were identified as containing the *vrg4*^+^ gene (SPAC144.18). The strains used in this study are listed in Table S1.

### Tagging the ***vrg4***^+^ gene

The upstream (genomic promoter) *vrg4*^+^ gene was amplified using primers Vrg4G-F (GGATCCCCCGGGTTGCGATGATGGTTTCAG) and Vrg4G-R (CGGTATCG ATAAGCTTTTATTTGTATAGTTCATCC). The *vrg4*^+^ gene and *GFP* gene were cloned using primers VRG4-F (ATGGATAATCATATGCTAA ACC) and UP-R (TATGATTATCCATTGTTGTTGGAATTTCGTG ATGG), GFP-F (ATGAGTAAAGGAGAAGAA) and GFP-R (TTATTTGTATAGTTCATCCA TG). Primers Down-F (GAACTATACAAATAAACCCTTTGTGTTGGTTATTA) and Down-R (GGTC GACGGTATCGATAAGCTTACAGTTCAAGCAACGAATT ACC) were used to amplify the downstream *vrg4*^+^ gene. These fragments were then cloned into a *pBC* + plasmid at *Sac* I and *Hind* III to generate the *pBC-vrg4*^+^*-GFP* plasmid. Then, the constructed *pBC-vrg4*^+^*-GFP* was used to transfect or construct site mutant plasmids. For site mutation plasmid construction, the following primers were used: S263C-F: (GGTGTGTACG CGTAACTTCTTGCACAACTTATAGTATG), S263C-R (CCAA CCATACTAT AAGTTGTGCAAGAAGTTACGCGTAC), A271V-F (CAACTTATAGTATGGTT GGTGTTCTTAACAAACTTC), A271V-R (CAAGGGAAGTTTGTTAAGAAC ACCAACCATACTATAAG), 263C-271 V-F (GTACGCGTAACTTCTTGCACAA CTTATAGTATGGTTGGTGTTCTTAACAA), and 263C-271 V-R (GGGAAGTTT GTTAAGAACACCAACCATACTATAAGTTGTGCAAGAAGTTA).

### Protein sequence alignment and structure simulation

The Vrg4 protein sequences of *S. cerevisiae* (KZV11004.1)*, S. pombe* (Q9UTK8) and *C. albicans* (AAK74075.1) were aligned in CLUSTALW. The protein topology structures of WT and mutant SpVrg4p proteins were predicted with the TMHMM Server v. 2.0 (http://www.cbs.dtu.dk/services/TMHMM/). The Vrg4 protein structure in *S. cerevisiae* (PDB 5OGK) was downloaded from the PDB protein data bank (http://www1.rcsb.org/) and used as a template to predict the structure of mutant SpVrg4p following the alignment mode procedure using Swiss-Model (https://swissmodel.expasy.org/)^19^. The WT Vrg4 (PDB 5OGK) and the produced mutant SpVrg4p structures were structurally aligned using TM-align (https://zhanglab.ccmb.med.umich.edu/TM-align/). All the PDB files were viewed in PyMOL v2.4 (https://pymol.org/2/), and the mutated residue sites are shown as sticks.

### Acid phosphatase assay

The details of the acid phosphatase assay method are described previously^20^. The cells were grown to mid-log phase at 27 °C in EMM. Then, the cells were collected, washed, resuspended in phosphate-free EMM, and incubated for 12 h at 27 °C to induce acid phosphatase. The cells were collected, washed once with 62.5 mM Tris–HCl (pH 6.8), and suspended in 240 ml of precooled lysis buffer (62.5 mM Tris–HCl, 1 mM EDTA, 2 mM phenylmethylsulfonyl fluoride, 0.1 mM dithiothreitol and 10% glycerol, pH 6.8). Cell lysates were prepared with 0.5 mM glass beads. The lysates were recovered and centrifuged at 15,000*g* for 10 min, and the supernatant was recovered and mixed with 1/3 volume of 0.1% bromophenol blue, 15% glycerol and 62.5 mM Tris–HCl (pH 6.8). Samples (10 μg protein) were loaded on 6% native polyacrylamide gel for electrophoresis. Electrophoresis and staining of the acid phosphatase activity were performed by the method described previously^21^.

### Immunoprecipitation and immunoblot analysis

Exponentially growing yeast cells (A600 = 1 ~ 3) were harvested and converted to spheroplasts as described previously^22^. The spheroplasts were resuspended in 400 ml of ice-cold lysis buffer (150 mM NaCl, 10 mM HEPES–KOH (pH 7.5), 5 mM MgCl2, 1 mM PMSF) containing 1% Triton X-100 to solubilize membrane proteins and centrifuged at 15 000 g for 5 min at 4 °C to remove debris. The detergent extracts were used in the coimmunoprecipitation assays. The GFP-tagged proteins were immunoprecipitated by incubating 400 ml of the extract with 200 ml of a hybridoma cell culture supernatant containing the monoclonal anti-GFP antibody and 25 ml of protein A Sepharose beads (Sangon Biotech) at room temperature for 4 h. The protein A Sepharose beads and associated proteins were centrifuged and washed three times with the same lysis buffer. After resuspension in sample buffer and solubilization at 45 °C for 3 min, the immunoprecipitates were separated by 10% SDS-PAGE, and gels between the markers containing our protein bands of interest were cut based on our previous experiment, transferred to PVDF membranes (Millipore) and immunoblotted with anti-GFP (1:2000) or anti-Got1p (1:2000) rabbit polyclonal antibody. Secondary rabbit antibodies conjugated to horseradish peroxidase (Sangon Biotech) were used at a 1:5000 dilution and detected by chemiluminescence (ECL, Thermo Scientific). All blotting was performed using a Tanon 2500 system (Shanghai Tanon Science and Technology Co., Ltd., Shanghai, China). All the unmodified data are shown in Supplementary Fig. 5.

## Results and discussion

### Isolation of *vas4-1* as a VPA-sensitive mutant

**Figure 1:**
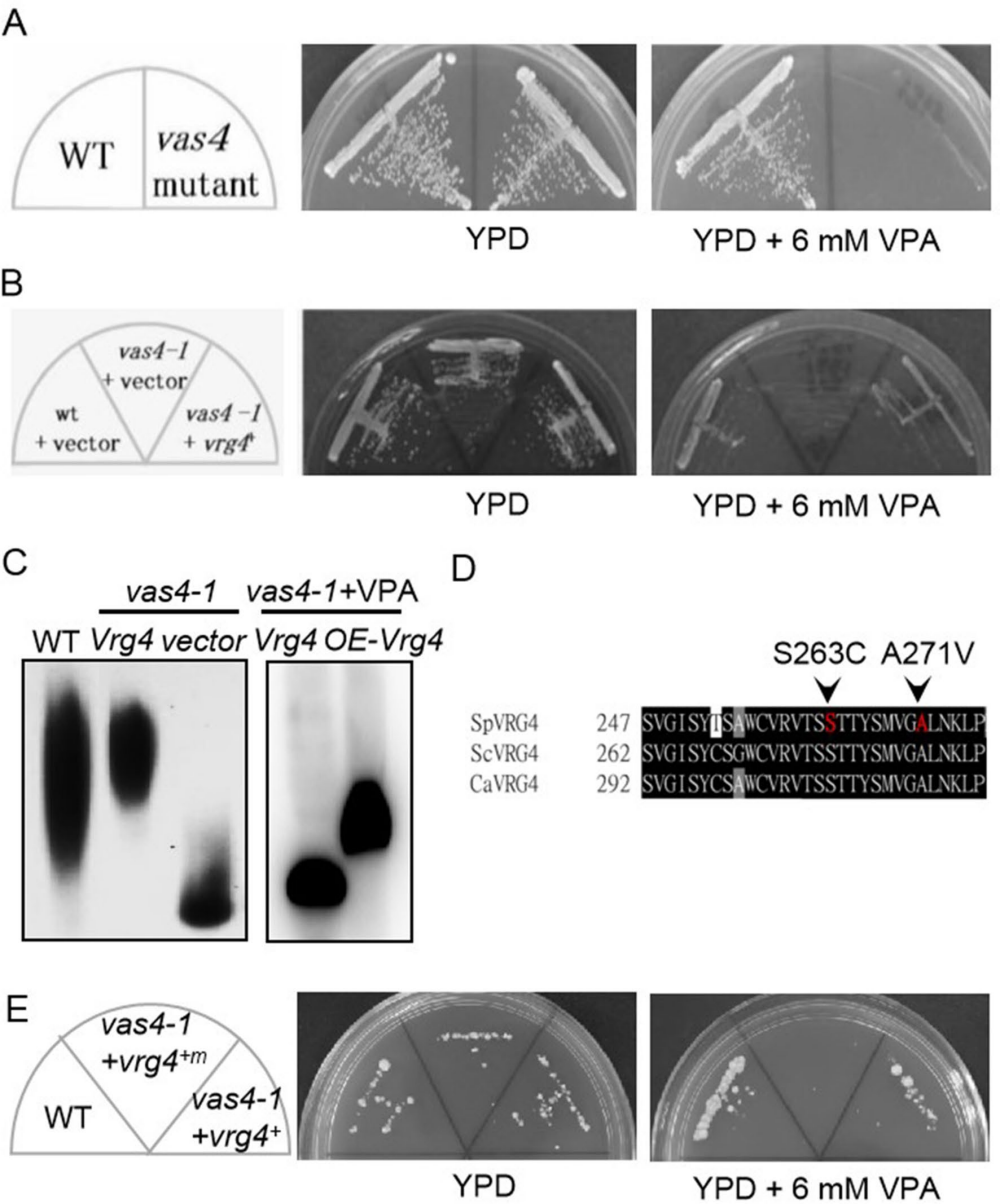
Mutation in the *vas4*^+^/*vrg4*^+^ gene leads to a VPA-sensitive phenotype. (**A**) *vas4-1* was identified as a VPA-sensitive mutant. WT and *vas4-1* were streaked on plates containing YPD or YPD plus 6 mM VPA and then incubated for 4 days at 27 °C. (**B**) The *vas4*^+^/*vrg4*^+^ gene can complement the VPA-sensitive phenotype of *vas4-1*. Cells transformed with control or a vector containing the *vas4*^+^/*vrg4*^+^ gene were streaked on plates containing YPD or YPD plus 6 mM VPA and then incubated for 4 days at 27 °C. (**C**) Acid phosphatase staining of cells with WT, *vas4-1* + Vector, *vas4-1* + *Vrg4*^+^, *vas4-1* + *Vrg4*^+^ + 6 mM VPA or *vas4-*1 + overexpressed (OE)*-Vrg4*^+^ + 6 mM VPA. (**D**) Partial protein sequence alignment of ScVrg4p, SpVrg4p and CaVrg4p. (**E**) S263C and A271V in SpVrg4p are the key contributors to VAP sensitivity. WT and *vas4-1* cells transformed with the control or a vector containing the *vas4*^+^/*vrg4*^+^ gene were streaked on plates containing YPD or YPD plus 6 mM VPA and then incubated for 4 days at 27 °C.

To identify genes that are involved in conferring sensitivity to VPA in *Schizosaccharomyces pombe* (*S. pombe*), we developed a genetic screen for valproic acid-sensitive (*vas*) mutants^10^. In the current study, we isolated and characterized a complementation group *vas4-1* mutant. As shown in Fig. 1A, the *vas4-1* mutant grew effectively, similar to the wild-type cells at 27 °C on yeast extract-peptone dextrose (YPD) plates, but the mutant failed to grow on a YPD plate containing 6 mM VPA at 27 °C.

### ***Vas4-1*** is an allele of the ***vrg4***^+^ gene that encodes a Golgi-located GDP-mannose transporter

**Figure 2:**
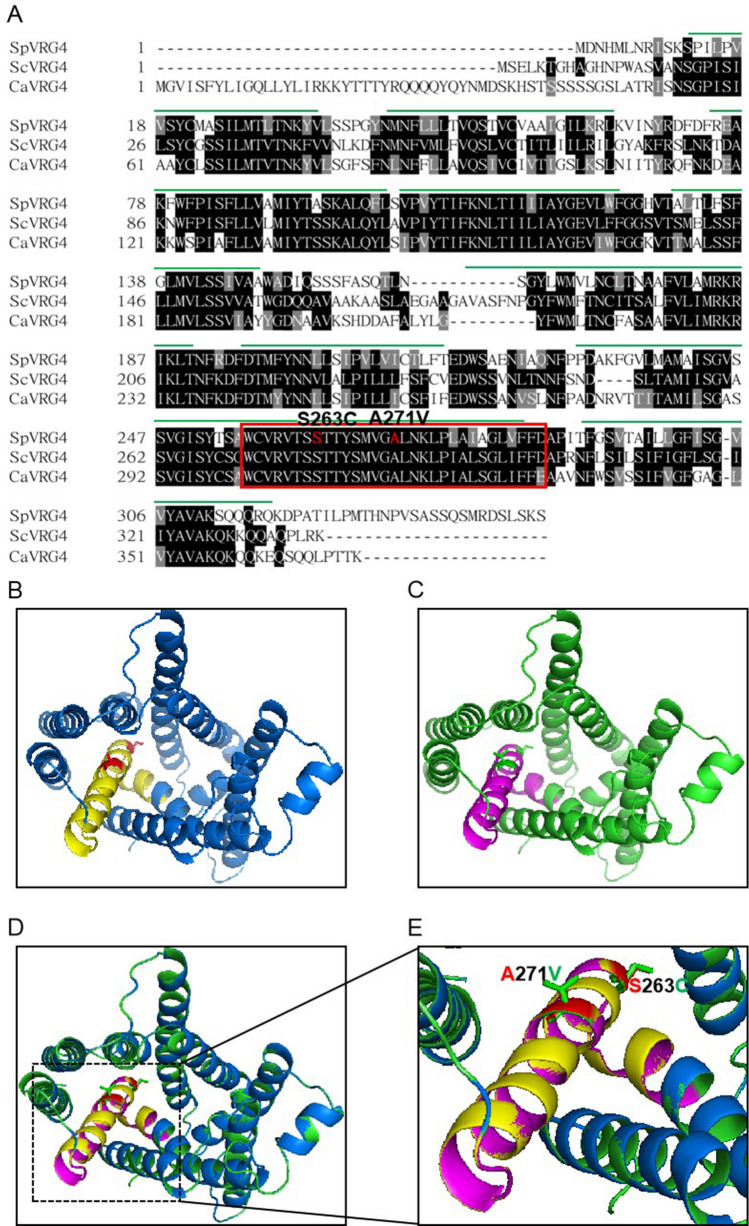
The S263C and A271V mutations had little effect on the overall structure of SpVrg4p. (**A**) Alignment of the Vrg4 protein sequences. The Vrg4 protein sequences of *S. cerevisiae, S. pombe and C. albicans* were aligned in CLUSTALW. The red orthogon indicates the reported nucleotide sugar-binding motif in ScVrg4p. The green lines represent the transmembrane helices in ScVrg4p. The mutation sites 263 and 271 are shown in red. (**B**)–(**E**). Golgi lumenal view of Vrg4p structures. WT (**B**), mutant (**C**), structural alignment (**D**) and closed views of aligned nucleotide sugar-binding motifs (**E**) are shown. The structure of mutant SpVrg4p was obtained from homologue modelling using Swiss-Model following the Alignment Mode procedure and structurally aligned with WT Vrg4p via TM alignment. The nucleotide sugar-binding motif is shown as yellow in the WT and magenta in the mutant protein. The mutated residue sites are shown in stick mode.

The *vas4*^+^ gene was cloned for complementation of the VAP-sensitive phenotype of the *vas4-1* mutant cells. Sequencing analysis showed that *vas4*^+^ was identical to the *vrg4*^+^ gene (SPAC144.18), which encodes a putative Golgi-located GDP-mannose transmembrane transporter. *vas4*^+^/*vrg4*^+^ encodes a protein of 345 amino acids that is highly similar to Vrg4p in *S. cerevisiae* (53.12% identity) and effectively complements the VAP-sensitive phenotype of *Vas4-1* (Fig. 1B). We next detected glycosylation by acid phosphatase staining of WT, *vas4-1* + vector and *vas4-1* + *vrg4*^+^ products. The results showed that *vas4-1* + *vrg4*^+^ had a glycosylation level similar to that of WT, while *vas4-1* + vector had an obviously lower glycosylation level (Fig. 1C). This result suggested that SpVrg4p also plays an important role in the glycosylation of fission yeast. VPA inhibited glycosylation in *vas4-1* + *vrg4*^+^ cells (Fig. 1C), and our results are in accordance with a previous report^10^. Glycosylation was, at least partially, recovered when VRG4 was overexpressed in *vas4-1* cells (Fig. 1C). This result indicated that VPA might target SpVrg4p and change the glycosylation pattern in *S. pombe*. The sequencing results indicated that *vas4-1* harboured two mutant sites (S263C and A271V) in the eighth transmembrane domain (Figs. 1D,2A). To explore whether the mutant sites in SpVrg4p were involved in the VAP-sensitive phenotype, the WT (*vrg4*^+^) and mutant harbouring the two mutant sites (*vrg4*^+*m*^) were introduced into *vas4-1* (Fig. 1E). A phenotype analysis indicated that *vrg4*^+^ and the two mutant sites were the key factors of VAP sensitivity.

### The S263C and A271V mutations had little effect on the overall structure of SpVrg4p

The function of Vrg4p in *S. cerevisiae* has been widely studied^14,15,23–26^. To explore the function of Vrg4p in fission yeast, we introduced mutations at sites A271V and S263C in SpVrg4p, which were coincidently consistent with previously reported ScVrg4p mutants *vig4-1* (A286V) and *vig4-2* (S278C)^23^ (Figs. 2A and S2A). *vig4-1* and *vig4-2* are expressed as vanadate resistant and immature glycosylation mutants in *S. cerevisiae*, suggesting that these two sites are important for the function of ScVrg4p^23^. The two mutation sites did not notably contribute to the predicted protein topology structure of SpVrg4p (Fig. S1). A protein domain analysis showed that these mutation sites are also located in a nucleotide sugar-binding motif^24^ (Fig. 2). The structure simulation, for which ScVrg4p (PDB: 5OGK) was the template^26^, indicated that the mutant amino acids had little effect on the overall and binding motif structures (Fig. 2B–E).

### SpVrg4p was important for maintaining cell wall integrity under temperature stress

To research the molecular function of SpVrg4p, WT and mutant Vrg4p-GFP plasmids were constructed and introduced into *vas4-1* mutant (Fig. S2A). We isolated consistent expression lines through Southern blotting, qRT-PCR and western blotting (Fig. S2B to D) and used them for subsequent research.

**Figure 3:**
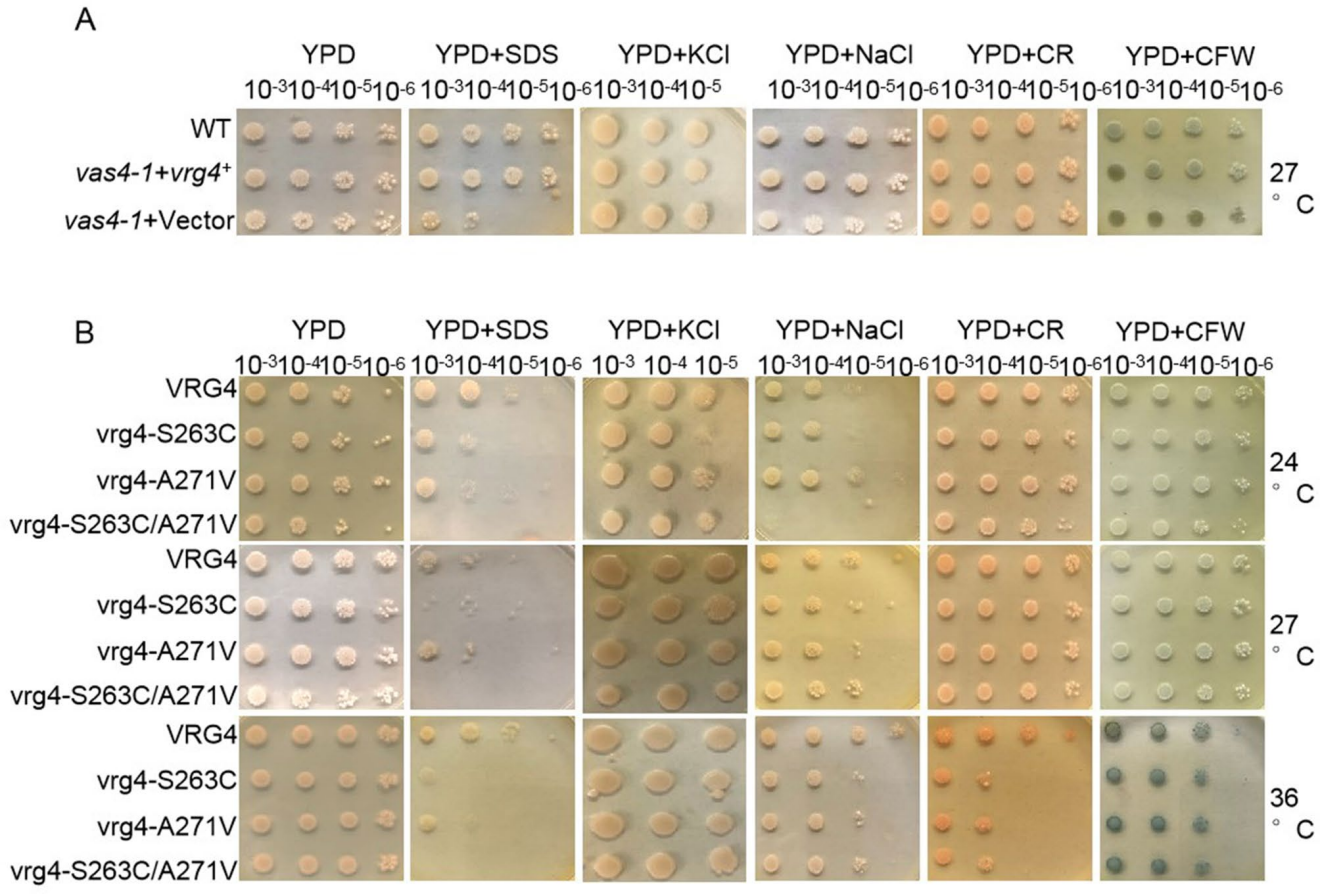
SpVrg4p is important for maintaining cell wall integrity under temperature stress. (**A**) WT and *vas4-1* cells transformed with the wild-type *vas4*^+^/*vrg4*^+^ gene or vector were incubated at 27 °C for 2 days. (**B**) *vas4-1* cells transformed with the WT or the mutant *vas4*^+^/*vrg4*^+^ gene were incubated at 24, 27, or 36 °C for 2 days. (**A**,**B**) Cells were spotted on plates containing YPD or YPD plus 100 μg/ml SDS, 1 M NaCl, 0.1% Congo red (CR) or 0.5 mg/ml calcofluor white (CFW), as indicated, in serial tenfold dilutions with the starting point indicated by OD_660_ = 0.3 in log-phase and then incubated for 2 days at the indicated temperature.

Previous studies accompanied by coexpression and interaction networks suggested that SpVrg4p is involved in protein glycosylation, cell wall integrity (CWI) and protein trafficking (Fig. S3)^14,23,25^. To reveal the function of Vrg4p in CWI in fission yeast, we first analysed WT, *vas4-1* + vector and *vas4-1* + *vrg4*^+^ cells grown with different CWI inhibition agents (Fig. 3A). The *vas4-1* + *vrg4*^+^ cells exhibited a growth phenotype similar to that of WT cells under various agent treatments, while the *vas4-1* + vector cells were more sensitive to SDS, NaCl and CFW. This result suggested that Vrg4p plays roles in maintaining CWI in fission yeast. To explore the function of the mutation sites in Vrg4p in CWI, the cell lines with consistent expression verified above were treated with CWI inhibitory agents at different temperatures. Cell viability was not obviously changed under high temperature stress, while the cells expressing the double mutant showed a slightly decreased growth rate at 24 °C (Fig. 3B column 1). At 27 °C, SDS treatment inhibited the growth of S263C and double mutant colonies, but other CWI inhibitory agents had no obvious effect. At 24 °C, SDS and NaCl treatment inhibited the growth of double mutant lines, while Congo red (CR) and calcofluor white (CFW) exerted slight effects on the double mutant lines (Fig. 3B row 1). Notably, both the single and double mutants exhibited obvious sensitivity to the CWI inhibitory agents at 36 °C (Fig. 3B row 3). These results suggested that SpVrg4p plays important roles in maintaining CWI under low- and high-temperature stress.

### Both single and double SpVrg4p mutants failed to interact with Got1p

**Figure 4:**
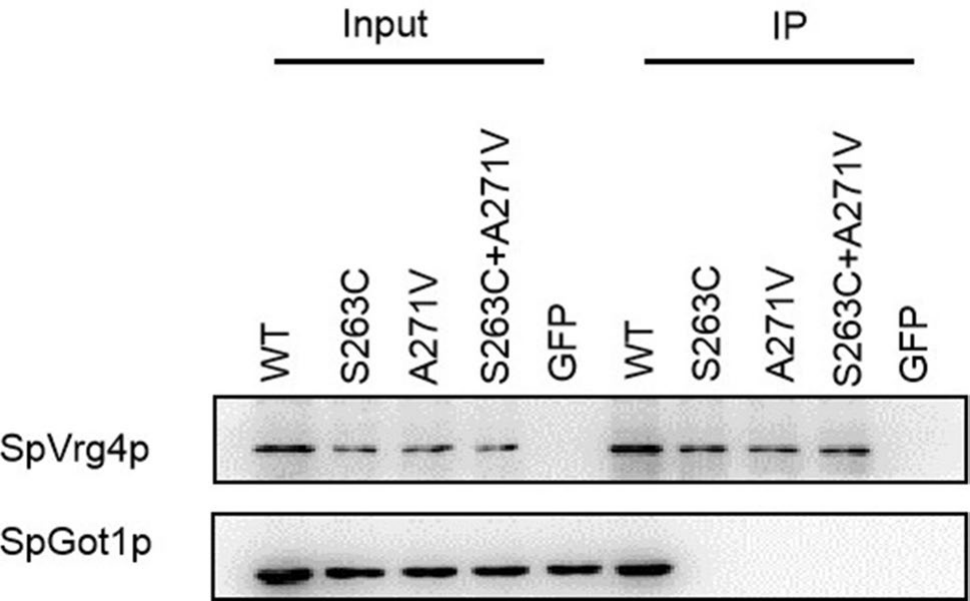
The mutations in SpVrg4p abrogated the interaction with Got1p. GFP and GFP-tagged Vrg4 were precipitated by GFP beads, washed extensively, subjected to SDS–PAGE and detected with anti-GFP or anti-Got1p antibody.

A previous study on ScVrg4p suggested that it is located in the Golgi, and the corresponding mutations of S263C and A271V in ScVrg4 had no obvious effect on its localization; however, the C-terminal region was determined to be essential for ScVrg4p Golgi localization^23^. Another study indicated that the *VRG4* gene is required for the retention of BiP in the ER, but it does not have an indirect effect by elevating the levels of misfolded proteins; the *vrg4* mutation affects the receptor-mediated retrieval of BiP from early Golgi^14^. Folded protein in the ER is transported to the Golgi through COPII vesicles. We found that Got1p, which is efficiently packaged into COPII vesicles and cycles rapidly between the ER and Golgi compartments^27^, was in the interaction network of SpVrg4p (Fig. S3). The coimmunoprecipitation assay indicated that SpVrg4 interacted with Got1p directly or directly (Fig. 4), while both the single and double (S263C and A271V) mutants abolished this interaction. These results suggested that SpVrg4p is transported from the ER to the Golgi by Got1p-loaded COPII vesicles and that the S263C and A271V mutations compromise this process. We tried to obtain GFP images; however, the results were ambiguous (data not shown). We found that overexpression of the mutant *vas4*^+*m*^ expression in isolated transgenic lines can partially recover the *vas4-1* VAP-sensitive phenotype (Fig. S4). These results were accordance to those previously reported^24^. Based on this result, the mutant might have little effect on the cellular localization of SpVrg4p. Interestingly, the S263C and A271V mutations had no obvious effect on the putative overall structure of SpVrg4p, but the mutants failed to interact with Got1p. SpVrg4p has 53.12% identity to *S. cerevisiae* Vrg4p, and although structure simulation may not present the true condition, it can provide certain information.

In summary, we isolated the VPA-sensitive mutant *vas4-1* and found that the *vas4*^+^/*vrg4*^+^ gene can complement the VPA-sensitive phenotype. The *vas4*^+^/*vrg4*^+^ gene may be a novel target of VPA. The *vas4*^+^/*vrg4*^+^ gene is important for CWI under temperature stress, and mutations at S263 and A271, which are located in the nucleotide sugar-binding motif, compromise Vrg4p function and abolish its interaction with Got1p. These results suggested that VPA might regulate physiological processes such as glycosylation and CWI through the *vas4*^+^/*vrg4*^+^ gene.

## Supplementary Information

Supplementary Information.

## References

[1] Johannessen CU, Johannessen SI (2003). Valproate: past, present, and future. CNS Drug Rev..

[2] Zhu MM, Li HL, Shi LH, Chen XP, Luo J, Zhang ZL (2017). The pharmacogenomics of valproic acid. J. Hum. Genet..

[3] Calabresi P, Galletti F, Rossi C, Sarchielli P, Cupini LM (2007). Antiepileptic drugs in migraine: from clinical aspects to cellular mechanisms. Trends Pharmacol. Sci..

[4] Johannessen CU (2000). Mechanisms of action of valproate: a commentatory. Neurochem. Int..

[5] Gurvich N, Klein PS (2002). Lithium and valproic acid: parallels and contrasts in diverse signaling contexts. Pharmacol. Ther..

[6] Catalano MG, Pugliese M, Poli R, Bosco O, Bertieri R, Fortunati N, Boccuzzi G (2009). Effects of the histone deacetylase inhibitor valproic acid on the sensitivity of anaplastic thyroid cancer cell lines to imatinib. Oncol. Rep..

[7] Chen X, Wong P, Radany E, Wong JY (2009). HDAC inhibitor, valproic acid, induces p53-dependent radiosensitization of colon cancer cells. Cancer Biother. Radiopharm..

[8] Schifitto G, Peterson DR, Zhong J, Ni H, Cruttenden K, Gaugh M, Gendelman HE, Boska M, Gelbard H (2006). Valproic acid adjunctive therapy for HIV-associated cognitive impairment: a first report. Neurology.

[9] Zhang XZ, Li XJ, Zhang HY (2010). Valproic acid as a promising agent to combat Alzheimer's disease. Brain Res. Bull..

[10] Miyatake M, Kuno T, Kita A, Katsura K, Takegawa K, Uno S, Nabata T, Sugiura R (2007). Valproic acid affects membrane trafficking and cell-wall integrity in fission yeast. Genetics.

[11] Ma Y, Takeuchi M, Sugiura R, Sio SO, Kuno T (2009). Deletion mutants of AP-1 adaptin subunits display distinct phenotypes in fission yeast. Genes Cells.

[12] Ma Y, Sugiura R, Zhang LL, Zhou X, Takeuchi M, He Y, Kuno T (2010). Isolation of a fission yeast mutant that is sensitive to valproic acid and defective in the gene encoding Ric1, a putative component of Ypt/Rab-specific GEF for Ryh1 GTPase. Mol. Genet Genomics.

[13] Zhang LL, Ma N, Liu QB, Ma Y (2013). Genome-wide screening for genes associated with valproic acid sensitivity in fission yeast. PLoS ONE.

[14] Poster JB, Dean N (1996). The yeast VRG4 gene is required for normal Golgi functions and defines a new family of related genes. J. Biol. Chem..

[15] Dean N, Zhang YB, Poster JB (1997). The VRG4 gene is required for GDP-mannose transport into the lumen of the Golgi in the yeast, *Saccharomyces cerevisiae*. J. Biol. Chem..

[16] Moreno S, Klar A, Nurse P (1991). Molecular genetic-analysis of fission yeast schizosaccharomyces-pombe. Method Enzymol..

[17] Zhang Y, Sugiura R, Lu Y, Asami M, Maeda T, Itoh T, Takenawa T, Shuntoh H, Kuno T (2000). Phosphatidylinositol 4-phosphate 5-kinase Its3 and calcineurin Ppb1 coordinately regulate cytokinesis in fission yeast. J. Biol. Chem..

[18] Beach D, Piper M, Nurse P (1982). Construction of a Schizosaccharomyces-Pombe gene bank in a yeast bacterial shuttle vector and its use to isolate genes by complementation. Mol. Gen. Genet..

[19] Waterhouse A, Bertoni M, Bienert S, Studer G, Tauriello G, Gumienny R, Heer FT, de Beer TAP, Rempfer C, Bordoli L, Lepore R, Schwede T (2018). SWISS-MODEL: homology modelling of protein structures and complexes. Nucleic Acids Res..

[20] Maeda T, Sugiura R, Kita A, Saito M, Deng L, He Y, Yabin L, Fujita Y, Takegawa K, Shuntoh H, Kuno T (2004). Pmr1, a P-type ATPase, and Pdt1, an Nramp homologue, cooperatively regulate cell morphogenesis in fission yeast: the importance of Mn2+ homeostasis. Genes Cells.

[21] Schweingruber AM, Schoenholzer F, Keller L, Schwaninger R, Trachsel H, Schweingruber ME (1986). Glycosylation and secretion of acid phosphatase in Schizosaccharomyces pombe. Eur. J. Biochem..

[22] Chi JH, Roos J, Dean N (1996). The OST4 gene of Saccharomyces cerevisiae encodes an unusually small protein required for normal levels of oligosaccharyltransferase activity. J. Biol. Chem..

[23] Abe M, Hashimoto H, Yoda K (1999). Molecular characterization of Vig4/Vrg4 GDP-mannose transporter of the yeast Saccharomyces cerevisiae. FEBS Lett..

[24] Gao XD, Nishikawa A, Dean N (2001). Identification of a conserved motif in the yeast golgi GDP-mannose transporter required for binding to nucleotide sugar. J. Biol. Chem..

[25] Hashimoto H, Abe M, Hirata A, Noda Y, Adachi H, Yoda K (2002). Progression of the stacked Golgi compartments in the yeast Saccharomyces cerevisiae by overproduction of GDP-mannose transporter. Yeast.

[26] Parker JL, Newstead S (2017). Structural basis of nucleotide sugar transport across the Golgi membrane. Nature.

[27] Lorente-Rodriguez A, Heidtman M, Barlowe C (2009). Multicopy suppressor analysis of thermosensitive YIP1 alleles implicates GOT1 in transport from the ER. J. Cell Sci..

