# Supplementary material for: Cell wall integrity is compromised under temperature stress in Schizosaccharomyces pombe expressing a valproic acid-sensitive vas4 mutant
Source: Sci Rep. 2021 Jun 29;11:13483. doi: 10.1038/s41598-021-92466-8 (PMC8242086; doi:10.1038/s41598-021-92466-8)
Supplement: Supplementary file 1 — Supplementary Information. [file 41598_2021_92466_MOESM1_ESM.docx]

**Supplementary Materials**

**
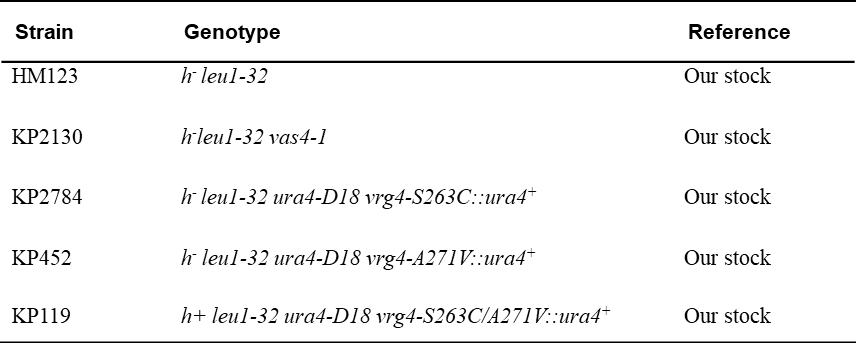
**

**Table. S1 *Schizosaccharomyces pombe* strains used in this study**


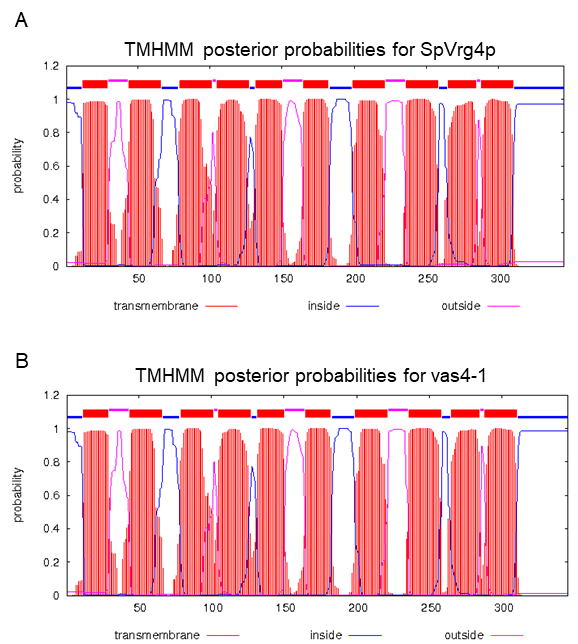


**Fig. S1 Predicted protein topology structures of WT and mutant SpVrg4p proteins**

The WT or mutant protein sequences were used to predicted the transmembrane helices in TMHMM Server v. 2.0 (http://www.cbs.dtu.dk/services/TMHMM/)


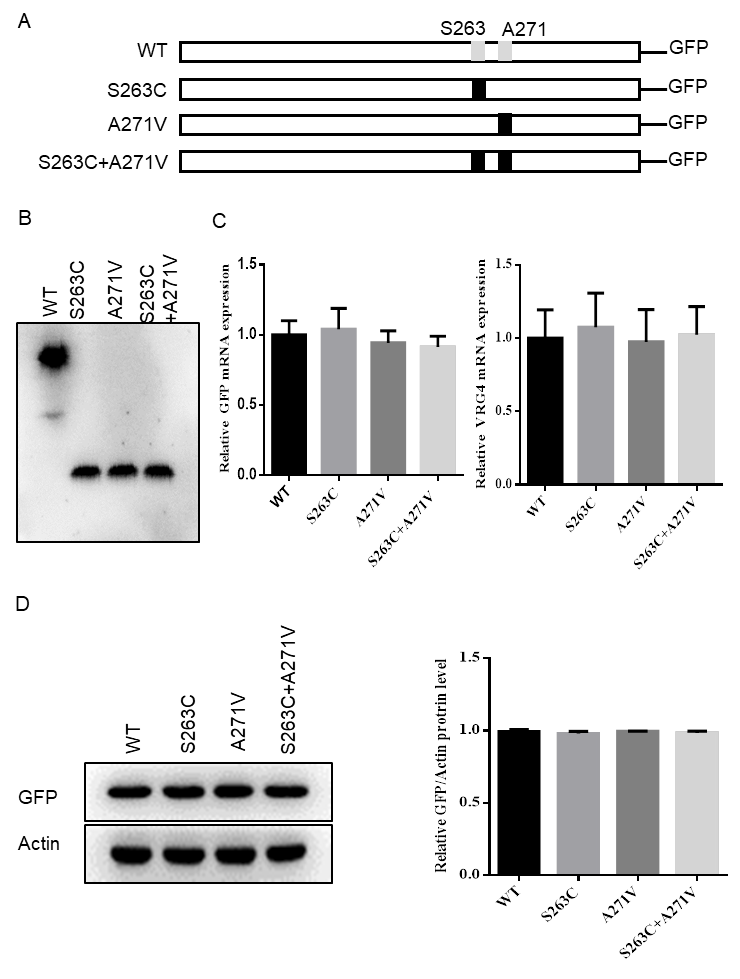


**Fig. S2 Isolated the consistent expression transformed lines**

A. Constructs. B. Southern blot of constructed vrg4 gene. C and D. The RNA and protein expression level of Vrg4-GFP in *vas4-1*.


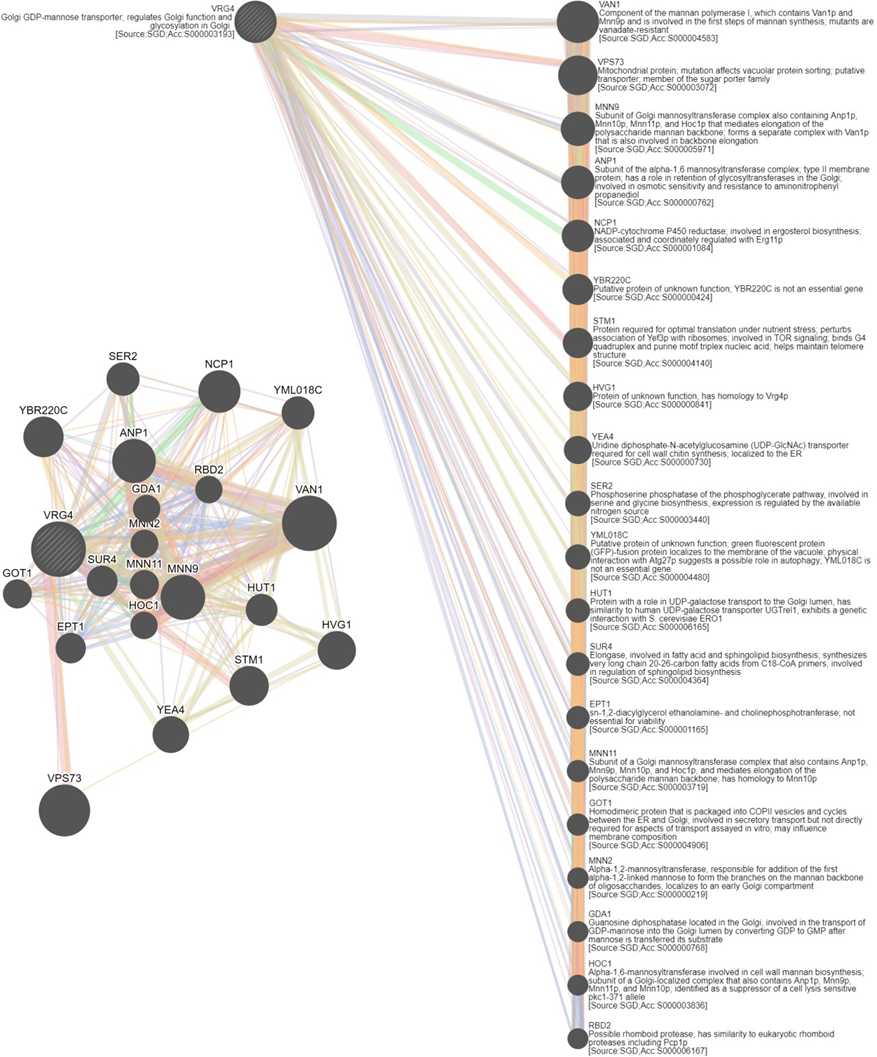


**Fig. S3 Co-expression and putative interaction network of Vrg4p**

The co-expression and interaction network of Vrg4p in GENEMANIA (http://genemania.org/search/saccharomyces-cerevisiae/vrg4)


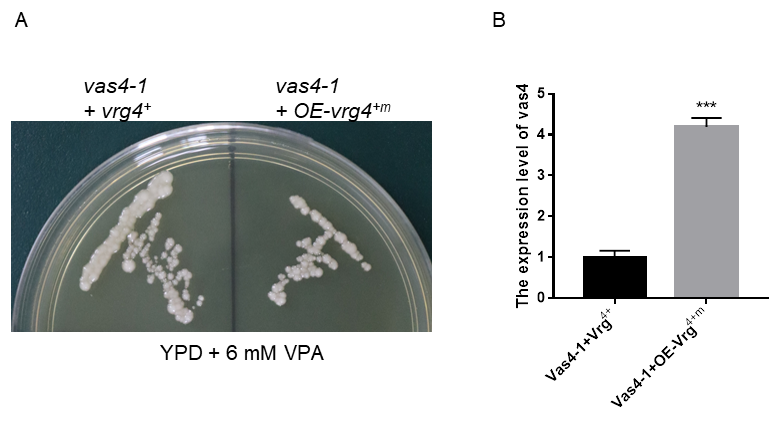


**Fig. S4 Overexpression the mutant *vas4^+m^* could partially recovery the *vas4-1* VAP sensitive phenotype**

A. *vas4* cells transformed with the vector containing the *vas4^+^/vrg4^+^* or *vas4^+m^* gene were streaked on plate containing YPD or YPD plus 6 mM VPA and then incubated for 5 days at 27℃. B. The expression level of *vas4^+^/vrg4^+^* or *vas4^+m^* in *vas4-1*.


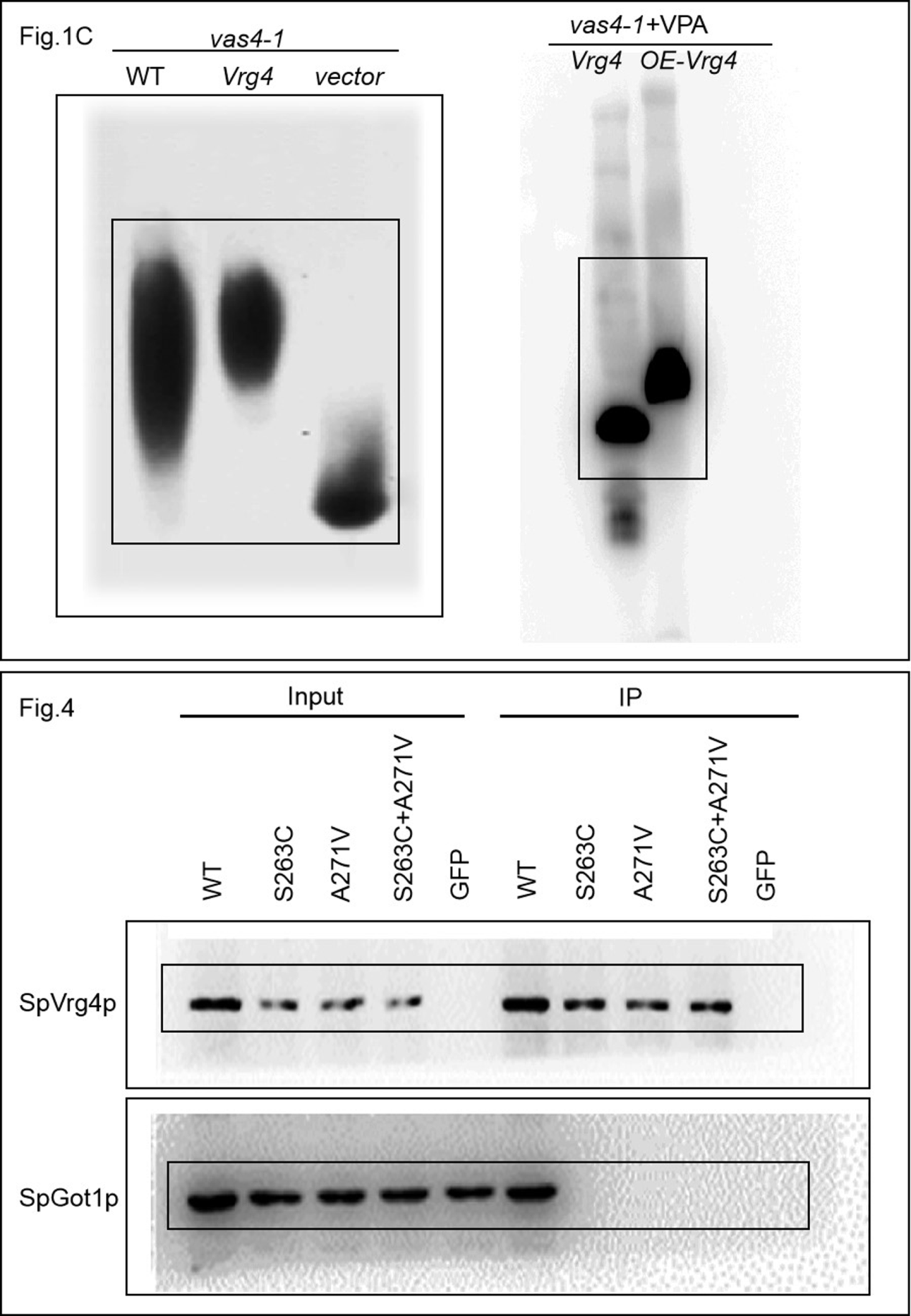


**Fig. S5a. Unmodified data. The unmodified illustrations used to prepare the main article.**

**
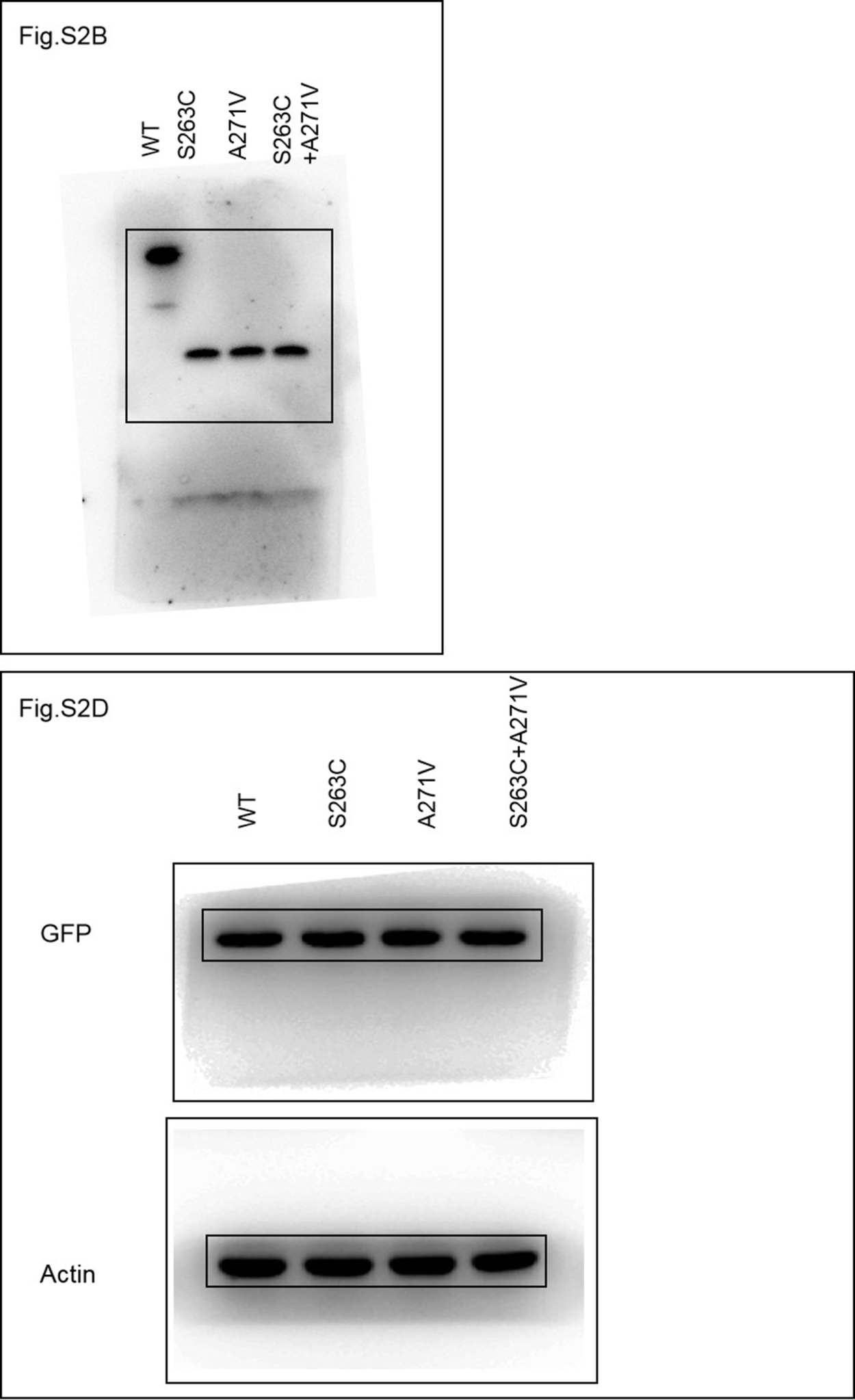
Fig. S5b. Unmodified data. The unmodified illustrations used to prepare the supplementary.**
